# S100A12 Serum Levels and PMN Counts Are Elevated in Childhood Systemic Vasculitides Especially Involving Proteinase 3 Specific Anti-neutrophil Cytoplasmic Antibodies

**DOI:** 10.3389/fped.2018.00341

**Published:** 2018-11-23

**Authors:** Kelly L. Brown, Joanna M. Lubieniecka, Giulia Armaroli, Katharina Kessel, Kristen M. Gibson, Jinko Graham, Dongmeng Liu, Robert E. W. Hancock, Colin J. Ross, Susanne M. Benseler, Raashid A. Luqmani, David A. Cabral, Dirk Foell, Christoph Kessel

**Affiliations:** ^1^Department of Pediatrics, University of British Columbia, Vancouver, BC, Canada; ^2^British Columbia Children's Hospital, Vancouver, BC, Canada; ^3^Department of Statistics and Actuarial Science, Simon Fraser University, Burnaby, BC, Canada; ^4^Department of Pediatric Rheumatology and Immunology, University Hospital Muenster, Muenster, Germany; ^5^Department of Medical Genetics, University of British Columbia, Vancouver, BC, Canada; ^6^Department of Microbiology and Immunology, University of British Columbia, Vancouver, BC, Canada; ^7^Centre for Microbial Diseases and Immunity Research, University of British Columbia, Vancouver, BC, Canada; ^8^Faculty of Pharmaceutical Sciences, University of British Columbia, Vancouver, BC, Canada; ^9^Department of Pediatrics, Alberta Children's Hospital, Calgary, AB, Canada; ^10^Nuffield Department of Orthopedics, Rheumatology and Musculoskeletal Sciences, University of Oxford, Oxford, United Kingdom

**Keywords:** chronic primary systemic vasculitis, neutrophils, proteinase 3, disease activity score, S100A12

## Abstract

**Objectives:** Chronic primary systemic vasculitidies (CPV) are a collection of rare diseases involving inflammation in blood vessels, often in multiple organs. CPV can affect adults and children and may be life- or organ-threatening. Treatments for adult CPV, although effective, have known severe potential toxicities; safety and efficacy of these drugs in pediatric patients is not fully understood. There is an unmet need for biologic measures to assess the level of disease activity and, in turn, inform treatment choices for stopping, starting, or modifying therapy. This observational study determines if S100 calcium-binding protein A12 (S100A12) and common inflammatory indicators are sensitive markers of disease activity in children and adolescents with CPV that could be used to inform a minimal effective dose of therapy.

**Methods:** Clinical data and sera were collected from 56 participants with CPV at study visits from diagnosis to remission. Serum concentrations of S100A12, C-reactive protein (CRP) and hemoglobin (Hb) as well as whole blood cell counts and erythrocyte sedimentation rate (ESR) were measured. Disease activity was inferred by physician's global assessment (PGA) and the pediatric vasculitis activity score (PVAS).

**Results:** Serum concentrations of standard markers of inflammation (ESR, CRP, Hb, absolute blood neutrophil count), and S100A12 track with clinically assessed disease activity. These measures—particularly neutrophil counts and sera concentrations of S100A12–had the most significant correlation with clinical scores of disease activity in those children with vasculitis that is associated with anti-neutrophil cytoplasmic antibodies (ANCA) against proteinase 3.

**Conclusions:** S100A12 and neutrophil counts should be considered in the assessment of disease activity in children with CPV particularly the most common forms of the disease that involve proteinase 3 ANCA.

**Key messages**:

- In children with chronic primary systemic vasculitis (CPV), classical measures of inflammation are not formally considered in scoring of disease activity.

- Inflammatory markers—specifically S100A12 and neutrophil count—track preferentially with the most common forms of childhood CPV which affect small to medium sized vessels and involve anti neutrophil cytoplasmic antibodies (ANCA) against proteinase-3.

## Introduction

Chronic primary systemic vasculitis (CPV) encompasses a group of rare and potentially life-threatening diseases characterized by inflammation in blood vessels in vital organs of the body (1). Systemic vasculitidies are classified according to their predominant involvement of large (e.g., Takayasu arteritis, TA), medium (e.g., polyarteritis nodosum, PAN), or small blood vessels [e.g., microscopic polyangitiis (MPA), granulomatosis with polyangiitis (GPA), and eosinophilic granulomatosis with polyangiitis (EGPA)]. Among the diseases with small vessel involvement, some are associated with anti-neutrophil cytoplasmic antibodies (ANCA). Individuals with MPA and GPA for example predominantly have circulating ANCAs against, respectively, the neutrophil proteins myeloperoxidase (MPO) and proteinase 3 (PR3) (2, 3). Clinically, PR3- and MPO-ANCA-associated vasculitis (PR3-AAV and MPO-AAV) are difficult to distinguish, but have some distinguishing features at least in adults; PR3-AAV often involves a greater number of organs and inflammation more frequently triggers necrotic lesions, whereas renal-limited disease occurs more often in patients with MPO-AAV (4). Data on AAV in pediatric populations are comparably scarce (2).

CPV can be treated with high-dose corticosteroids and cyclophosphamide, but these treatments have significant adverse effects (1, 3). Novel therapeutic options include biologics that target specific immune cells (such as anti-CD20 monoclonal antibody) and immune-cell derived cytokines such as TNFα, IL6 and IL1β. Balancing the risks associated with existing or emerging treatments against the damage associated with either under- or over-treating disease is an ongoing clinical challenge (5–9). In addition, despite advances in treatment, most patients suffer a chronic or relapsing disease course especially in the initial years following diagnosis. In individual patients, the ability to assess the presence, absence, and level of inflammatory activity across a range of organs, and distinguish this from prior damage is challenging—yet critical—in determining when to stop, start, or modify treatment. Delivery of such personalized therapy requires reliable measures of whether the disease is responsive to treatment (improving or in remission), refractory to treatment, or relapsing after previous improvement or remission.

Several tools have been developed to assess disease activity in adults with AAV by systematic evaluation of the contribution of different organ systems to disease activity. Most of these clinical tools derive from the Birmingham Vasculitis Activity Score (BVAS) which is a validated and widely used tool established for use in clinical trials for adult AAV (10, 11). A pediatric adaptation of BVAS, the pediatric vasculitis activity score (PVAS) has been preliminarily validated (12). Both BVAS and PVAS, however, have inherent limitations in particular in the ability to assess low levels of inflammation; the inferred absence of disease activity as scored by BVAS/PVAS often does not correlate with the underlying biological processes being switched off—as evidenced by patients frequently experiencing relapse despite a score indicating inactive disease. Another limitation to PVAS is practitioner preference and familiarity with a visual analog scale to score overall disease activity (Physician's Global Assessment; PGA) (13).

Both BVAS and PVAS are based on the assessment of clinical features of vasculitis and do not consider routine laboratory markers of inflammation, such as erythrocyte sedimentation rate (ESR) and C-reactive protein (CRP). Use of such measures in tracking vasculitis-related inflammation in adults beyond diagnosis is generally considered controversial, in part because ESR and CRP are affected by renal function, high dose corticosteroids, concurrent infections and other co-morbidities (14, 15). In contrast and despite not being formally scored in PVAS, ESR, and CRP are routinely measured and considered in the clinical evaluation of disease activity in childhood CPV (16). Yet, the relative utility of clinical tools and laboratory markers of inflammation have not been evaluated in pediatric CPV. We and others previously established that S100 calcium-binding protein A12 (calgranulin C, S100A12), an intrinsic protein (17) released by activated neutrophils, is a reliable marker of systemic inflammation (13) that tracks with disease activity in an acute form of childhood vasculitis (18, 19). Now, in this observational study our aim was to determine the potential clinical utility of S100A12 and inflammatory markers currently measured in clinical practice (Hemoglobin (Hb), ESR, CRP, absolute cell counts) for the improved asessment of disease activity in pediatric CPV.

## Materials and methods

### Study participants

Eligibility criteria for the Pediatric Vasculitis (PedVas) study have been described previously (20); for this study, written informed consent was obtained between April 2013 and October 2014 from a total of 56 children or adolescents 18 years of age and under (range 2–17 year) with chronic systemic primary vasculitis (CPV). The study protocol was approved by the Children's and Women's Research Ethics Board of the University of British Columbia (H12-00894) and the respective ethical committees or IRBs at participating PedVas study sites at 10 institutions in Canada (*n* = 3), the United States (*n* = 4), and Europe/Asia (*n* = 3).

### Study visits

PedVas study visits were designed to coincide with times of standard clinical care, namely at diagnosis (D), post-induction therapy (PTI; 3–6 months after diagnosis when induction therapy is complete), one-year follow-up (FU; 12 months post diagnosis (range 11–15 months), flare (F; ≥18 months post diagnosis with a change in a major PVAS score and sustained escalation of treatment), and remission (R; ≥18 months from diagnosis and PVAS = 0). Participants were eligible to enter the study at any visit and donated clinical data and blood at all study visits that occurred between April 2013 and Oct 2014.

### Clinical data

The clinical data included demographics, clinical features, medical history, diagnostic data, treatment and basic clinical laboratory results. This data set was described previously (21) and data was entered by participating sites into ARChiVE (A Registry of Childhood Vasculitis), a database for chronic pediatric systemic vasculitis that is housed on a secure, web-based application called RedCap (22) and hosted at the University of British Columbia. Clinical data from participating sites was reviewed in Vancouver for omissions and errors, and complete data was locked.

### Classification

Pediatric CPV was diagnosed and classified by the pediatric rheumatologist at participating sites (Table 1) and include granulomatosis with polyangiitis (GPA, formerly Wegener's granulomatosis, *n* = 20), microscopic polyangiitis (MPA, *n* = 4), eosinophilic granulomatosis with polyangiitis (EGPA, formerly Churg-Strauss syndrome, *n* = 4), and renal-limited pauci-immune glomerulonephritis (*n* = 2), polyarteritis nodosa (PAN, *n* = 6), cutaneous PAN (*n* = 3), Takayasu's arteritis (TA, *n* = 14), and unclassified vasculitis (UCV, *n* = 3).

**Table 1:** Study samples.

**Classification of vasculitis**	**Patients (*n* = 56)/Samples (*n* = 117)**	**Age (years) at Dx median (range)**	Study visit^*^				
			**D**	**PTI**	**FU**	**F**	**R**
Microscopic polyangiitis (MPA)	4/7	17 (14–19)	3	2	2	1	–
Granulomatosis with Polyangiitis (GPA)	22/46	13 (4–18)	17	11	11	4	4
Eosinophilic Granulomatosis with Polyangiitis (EGPA)	4/10	17 (10–17)	3	3	3	–	1
ANCA-Glomerulonephritis (ANCA-GN)	2/4	16	1	2	1	–	–
Polyarteritis Nodosa (PAN) including cutaneous PAN	9/15	5.5 (4–12)	4	4	2	2	3
Takayasu's Arteritis (TA)	15/27	13 (4–17)	11	–	7	–	1
Unclassified Vasculitis (UCV)	1/3	10	1	1	1	–	–

* *indicates number of patients providing samples at individual study visits*.

*D, diagnosis; PTI, post-therapy induction; FU, 12-month follow-up; F, flare; R, remission*.

### Measures of disease activity

The on-site rheumatologist provided a physician's global assessment (PGA) of overall disease activity; this is a subjective numeric value scored on a 10 cm visual analog scale. A PVAS was generated from data entered into the registry by participating sites and is based on a combined weighted scoring of clinical measures that probe the health of each organ system (12). As neither PVAS or PGA is a gold standard for assessment of disease activity, activity was also inferred based on the study visit with samples drawn at diagnosis and flare being most likely to reflect active disease. Conversely, samples > 18 months post diagnosis with a PVAS score = 0 are most likely to reflect inactive disease. Study visits in between likely correspond to increasingly mild disease moving from post-therapy induction to 12-months post diagnosis.

### Whole blood and sera

Blood (117 samples from 56 patients) was collected at participating centers. As part of routine clinical care, complete blood cell counts (CBC), Hb concentration and ESR were performed at participating sites. Processed blood sera was shipped to Vancouver and stored in aliquots at−80°C for CRP and S100A12 analysis. Normative ranges for healthy children were determined by Muenster University Hospital: absolute polymorphonuclear leukocytes (PMN count) 2–6 × 10^9^ cells/L, sera concentration of CRP (high sensitivity assay) < 0.5 mg/dl, erythrocyte sedimentation rate (ESR) 3-18 mm/h, and sera concentrations of hemoglobin (Hb) 114–143 g/L.

### Immunoassays for CRP, S100A12, and ANCA

Concentration of CRP (high-sensitivity CRP, DCRP00, R&D Systems) was measured in participants sera by ELISA according to manufacturer's instructions. As methods for ANCA testing differ at participating sites, patient sera was re-analyzed using a standard ELISA for anti-PR3 antibody (ORG518, Orgentec) and anti-MPO antibody (425–2,380, BioRad) according to manufacturer's instructions. Concentration of S100A12 in sera from participants was measured with an in-house monoclonal antibody sandwich ELISA directed against a defined epitope on S100A12; samples from 32 healthy children and adolescents established that normal serum concentrations of S100A12 are < 75 ng/ml.

### Statistical analysis

Multiple group comparisons were performed by Kruskal-Wallis test followed by Dunn's multiple comparison analysis (GraphPad Prism software (Version 6.0 for Mac OS X, GraphPad Software, La Jolla, CA, United States). *p* < 0.05 was considered statistically significant. Spearman correlation following Bonferroni correction for multiple correlations of laboratory markers with PVAS and PGA as well as with each other was performed for those participants with complete and paired clinical and laboratory data (ANCA negative: *n* = 17 samples; MPO-AAV: *n* = 20 samples; PR3-AAV: *n* = 15 samples). These were calcuated in a pair-wise comparison and plotted using the corrplot R package and Rstudio (23).

## Results

### S100A12, ESR, CRP, and Hb track with childhood CPV disease activity

To determine if common indicators of inflammation (blood cell counts, ESR, CRP, Hb) and S100A12 correlate with disease activity over the course of CPV, we analyzed a total of 117 serum samples obtained concurrent with clinical data from 56 children with systemic vasculitis; 48 samples were collected at times likely to correspond to high disease activity (diagnosis and flare), and 69 samples at follow up visits when disease activity should be reduced. Of the 56 children, 21 had samples collected from only one study visit, 9 had samples collected from two study visits, and 26 children had samples collected from 3 or more study visits.

**Figure 1:**
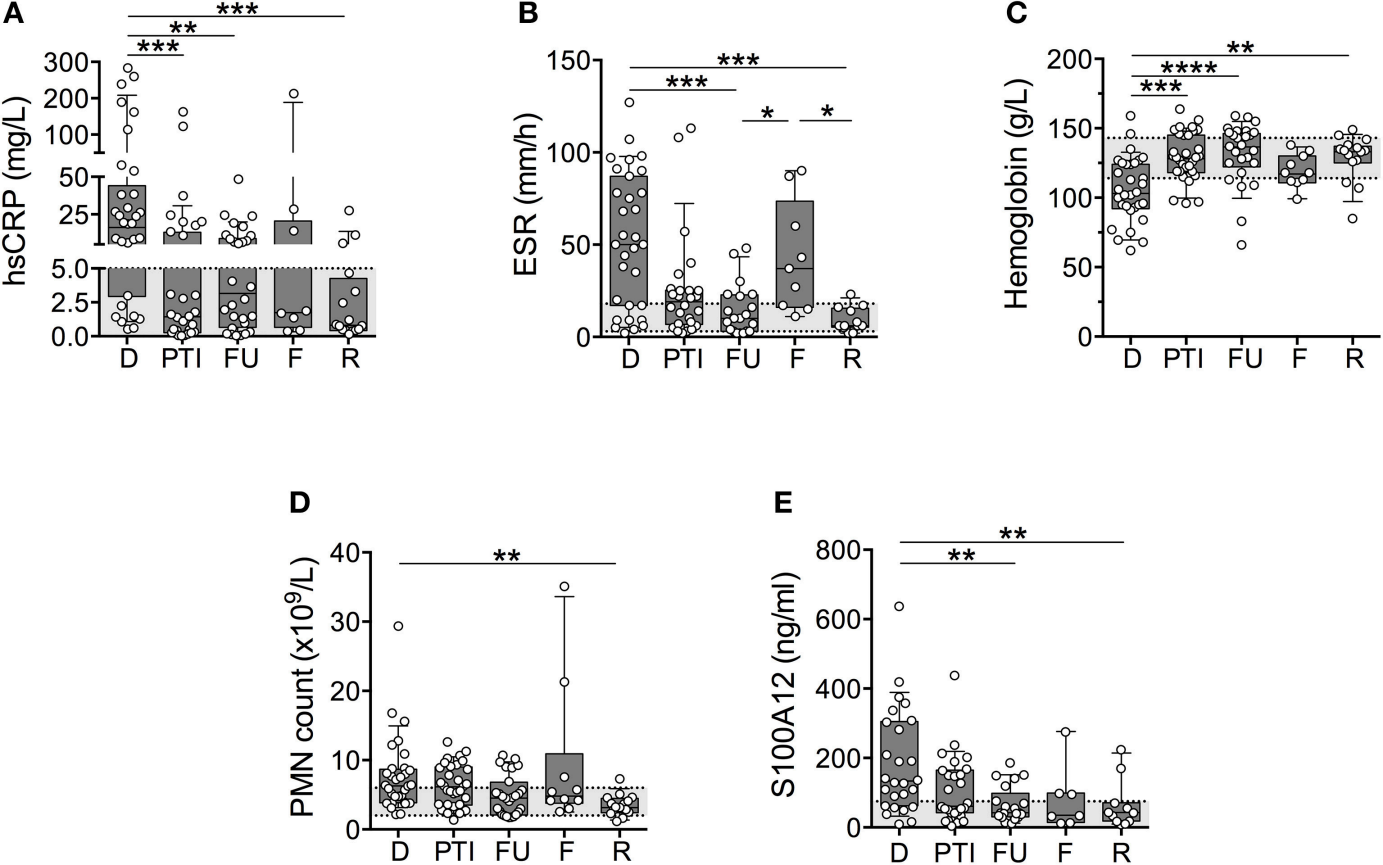
Common markers of inflammation measured in a cohort of children with systemic primary vasculitis. Serum and whole blood samples (*n* = 117) obtained from pediatric vasculitis patients (*N* = 56) over the course of disease (x-axis) including diagnosis (D), post induction therapy (PTI), one year follow-up (FU), flare (F), and remission (R) were analyzed (y-axis; log or linear (C) scale) for **(A)** concentrations of C-reactive protein (CRP (mg/dl), *n* = 77), **(B)** erythrocyte sedimentation rate (ESR (mm/h), *n* = 74), **(C)** concentrations of Hb (g/L, *n* = 80), **(D)** absolute polymorphonuclear leukocytes (PMN count × 10^9^/L, *n* = 79), and **(E)** concentration of S100A12 (ng/ml, *n* = 93). Respective normal ranges in gray shading for healthy children 4–20 years of age (see Materials and Methods). Data were analyzed by Kruskal-Wallis test followed by Dunn's multiple comparison test. Data are presented as box and whisker (10–90th percentile) with over-layed scattered dot plots ± SEM with means shown as horizontal lines. *p < 0.05, **p < 0.01, ***p < 0.001, and ****p < 0.0001.

ESR and concentrations of S100A12, CRP, and Hb fell outside of pediatric normal ranges (Figures 1A–E) most dramatically at the onset of disease (diagnosis). Measured values of S100A12, ESR and CRP were abnormally high whereas Hb was abnormally low for the majority of patients (mean values: CRP = 47 mg/L, ESR = 53 mm/h, Hb = 105 g/L, S100A12 = 247 ng/mL). Concentrations of Hb normalized for the majority of patients following 3-6 months of induction therapy (Figure 1C), and remained within or close to the normal range at follow up visits (at remission, mean concentration of Hb = 128 g/L). Measures of CRP, ESR, and S100A12 normalized for many patients following 3–6 months of induction therapy and substantially more patients at 12-months post diagnosis (Figures 1A,B,E). CRP, ESR and S100A12 were within the normal range for the majority of patients at remission (mean values: CRP = 3.4 mg/L, ESR = 9.3 mm/h, S100A12 = 64 ng/mL) and spiked during times of disease flare most notably for ESR and CRP (Figures 1A,B). Absolute blood neutrophil (PMN) counts were significantly elevated at diagnosis and flare compared to samples collected during remission; otherwise no other significant changes in patients' PMN-counts over the course of disease were observed (Figure 1D).

To decipher if laboratory markers could accurately track with disease activity for individual patients over the course of disease (as compared to cohort averages), we selected individual patients (N) with samples from at least 3 study visits for analysis of CRP (*N* = 13), ESR (*N* = 12), Hb (*N* = 15), PMN (*N* = 15), and S100A12 (*N* = 10). These data (not shown) revealed that changes in sera concentrations of CRP (*N* = 6/13) and ESR (*N* = 5/12) in approximately half of the individuals were consistent with trends observed for the cohort. Notably, measures of Hb (*N* = 11/15, 73%), S100A12 (*N* = 7/10, 70%), and PMN counts (*N* = 9/15, 60%) in the majority of individual patients mirrored changes in the larger cohort.

### Serum S100A12 and PMN counts are elevated in PR3-AAV

To determine if inflammatory indicators tracked similarly in patients differing in ANCA, patients were stratified for ANCA-positivity and specificity based on site-reported ANCA testing at diagnosis and subsequent in-house validation using standard anti-MPO and anti-PR3 immunoassays (data not shown). Of the 44 patients with small to medium-sized vessel vasculitis (GPA, MPA, EGPA, PAN, and unclassified vasculitis), 15 patients (predominantly PAN) were negative for ANCA, 13 patients (exclusively GPA) had ANCA toward PR3 (PR3-AAV), and 14 patients (inclusive of all MPA) had ANCA toward MPO (MPO-AAV).

**Figure 2:**
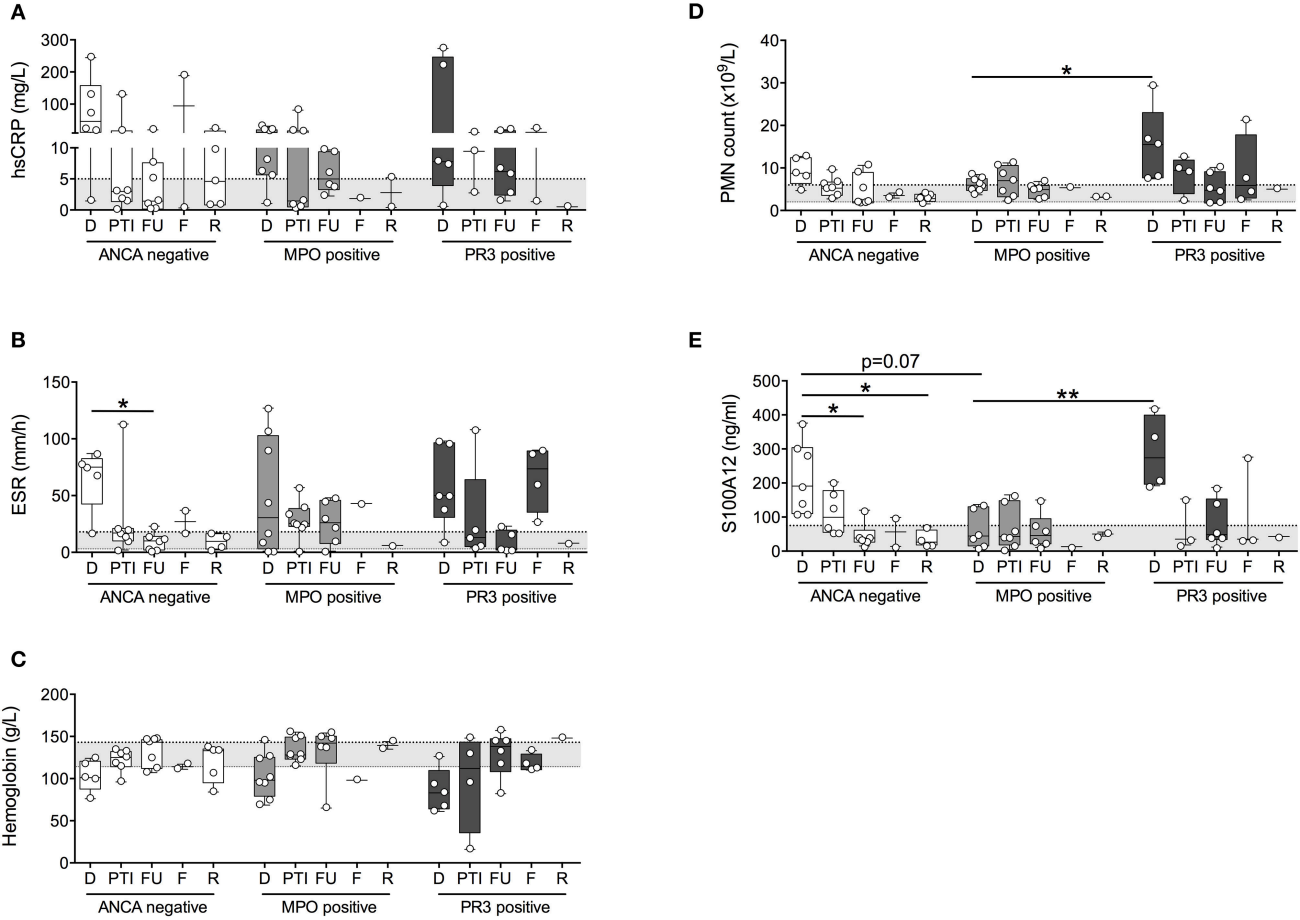
Measures of inflammation stratified according to ANCA status. Patients with small to medium sized vessel vasculitis were stratified for ANCA-status (x-axis; ANCA-negative, MPO-positive, PR3-positive) and blood samples collected at diagnosis (D), post therapy induction (PTI), one year follow-up (FU), flare (F), and remission (R) were analyzed [y-axis, log and linear (C) scale] for **(A)** concentrations of C-reactive protein (CRP (mg/dl), **(B)** erythrocyte sedimentation rate (ESR (mm/h)), **(C)** concentrations of Hb (g/L), **(D)** absolute polymorphonuclear leukocytes (PMN count × 10^3^/ul), as well as **(E)** concentration of S100A12 (ng/ml). Respective normal ranges in gray shading for healthy children 4–20 years of age (see Materials and Methods). Data were analyzed by Kruskal-Wallis test followed by Dunn's multiple comparison test. Data are presented as box and whisker (10–90th percentile) with over-layed scattered dot plots ± SEM with means shown as horizontal lines. *p < 0.05 and **p < 0.01.

Similar to findings presented in Figure 1, inflammatory indicators tracked with disease activity yet this was preferential to patients with ANCA-negative vasculitis and PR3-AAV (Figures 2A–E). While Hb concentration (Figure 2C) tracked with disease activity in MPO-AAV, other inflammatory markers were generally in the normal range and/or varied little between study visits that capture times of varying disease activity. This was particularly notably for absolute blood PMN counts and serum concentrations of S100A12 that were significantly elevated at the time of diagnosis in PR3-AAV compared to measures in patients with MPO-AAV that, for the most part, were within the normative range (Figures 2D,E).

**Figure 3:**
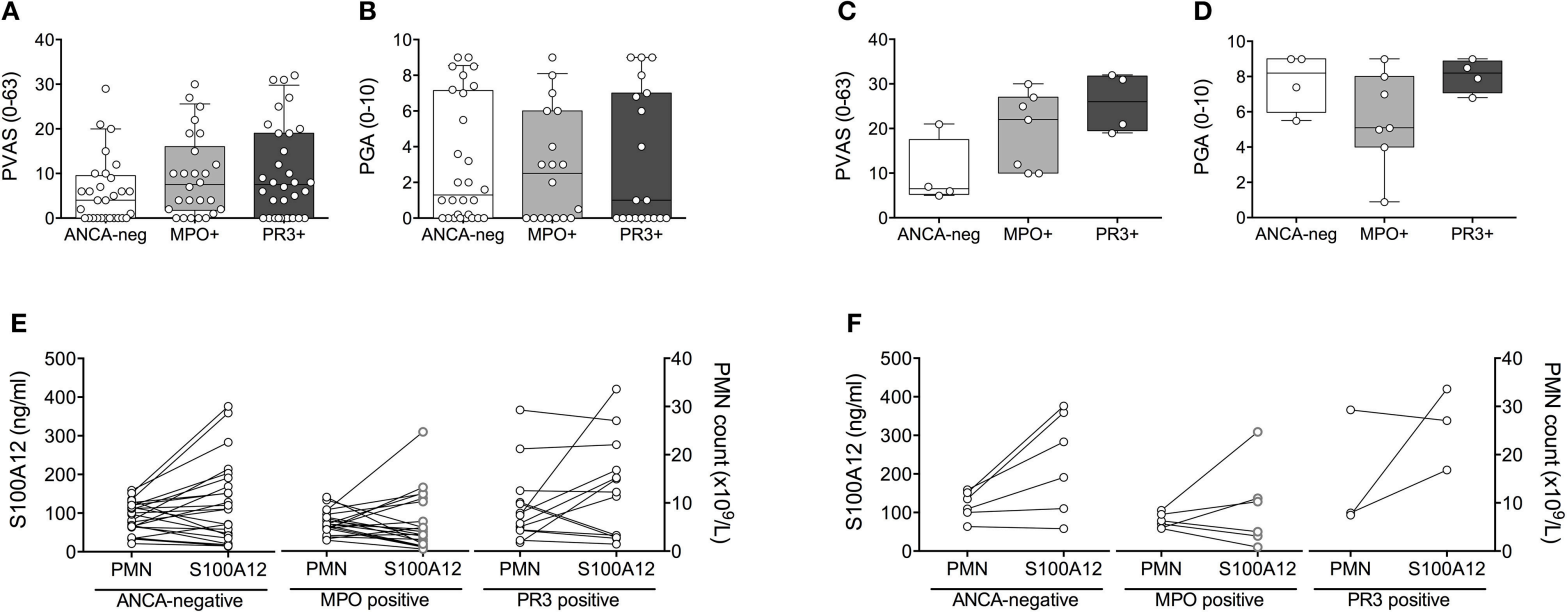
Clinical disease activity scores and impact of corticosteroid treatment relative to ANCA-status. Clinical disease activity scores **(A,C)** PVAS (y-axis, range 0–63) and **(B,D)** PGA (y-axis; range 1–10) for children with small to medium sized vessel vasculitis that is associated (x-axis) with the absence (ANCA-neg) or presence of either MPO-ANCA (MPO+) or PR3-ANCA (PR3+) is shown for all study visits **(A,B)** and at the time of diagnosis **(C,D)**. Data **(A–D)** were analyzed by Kruskal-Wallis test followed by Dunn's multiple comparison test. Data are presented as box and whisker (10–90th percentile) with over-layed scattered dot plots ± SEM with means shown as horizontal lines. **(E,F)** Absolute polymorphonuclear leukocytes (**E,F**; right y-axis, PMN count × 10^9^/L), and related concentration of S100A12 (**E,F**; left y-axis, ng/ml) in patients with varying ANCA status (x-axis) receiving corticosteroid treatment over the course of disease **(E)** or specifically at the time of diagnosis **(F)** is shown.

The observed elevation in PMN and S100A12 in patients with PR3-AAV and, to a lesser extent, ANCA-negative vasculitis affecting small to medium vessels, was not attributable to higher disease activity in these individuals. PVAS and PGA did not differ significantly between patients with ANCA-negative vasculitis, MPO-AAV or PR3-AAV over the course of disease (Figures 3A,B) or specifically at the time of diagnosis (Figures 3C,D). Moreover, results do not appear to be related to receipt of corticosteroid treatment; although treatment in our study cohort was heterogenous, we observe no significant difference in PMN counts or related S100A12 concentration in patients receiving corticosteroid (CS) therapy over the course of disease (Figure 3E) or specifically at the time of diagnosis (Figure 3F).

### Clinical disease activity scores PVAS and PGA correlate with inflammatory markers in PR3-AAV

**Figure 4:**
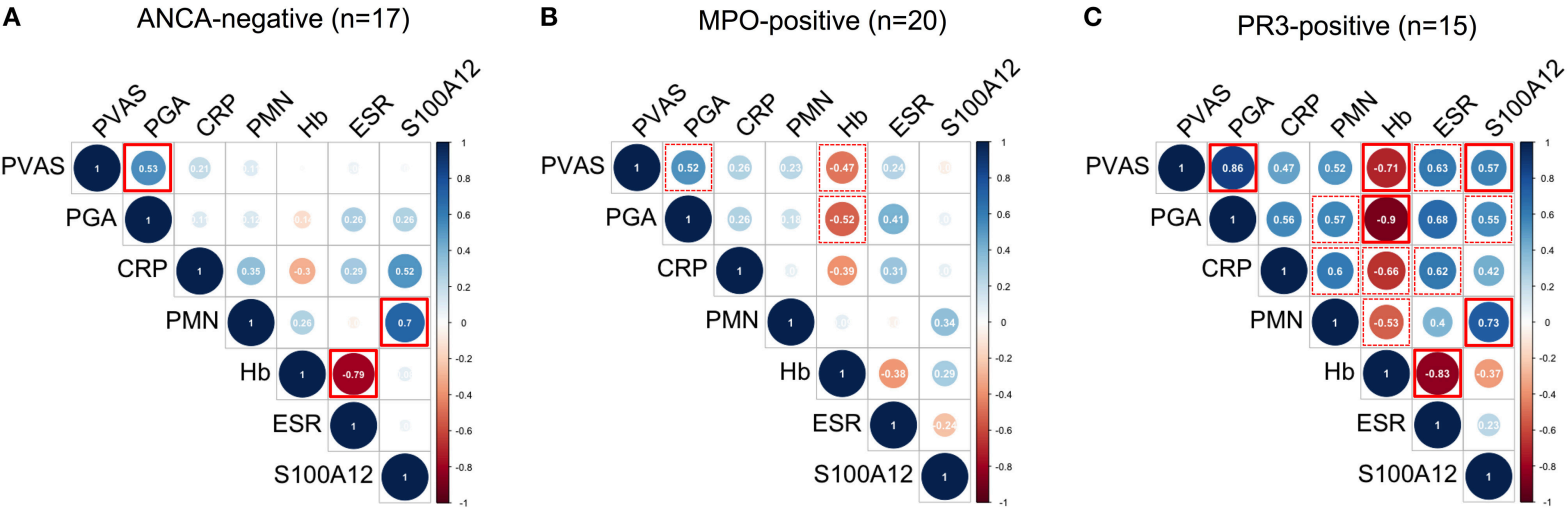
Multiple correlation analyses of laboratory markers and clinical disease activity scores. Correlograms show spearman correlations of clinical disease activity scores (PVAS and PGA) with quantified measures of inflammation: concentration of C-reactive protein (CRP), erythrocyte sedimentation rate (ESR), concentration of hemoglobin (Hb), absolute polymorphonuclear leukocyte (PMN) count, and concentration of S100A12 in samples (n) obtained from ANCA-negative **(A)** MPO-positive **(B)** and PR3-positive **(C)** CPV patients. Color indicates positive (blue) and negative (red) correlation. Color intensity and circle diameter indicates strength of the correlation (red: −1 to blue: +1). Dash red boxes indicate significant correlations, red boxes with full lines indicate significant correlations following Holm-Bonferroni correction for multiple correlation analyses.

Finally, we performed multiple correlation analyses in the ANCA-stratified cohorts on quantified measures of inflammation and disease activity scores currently used in clinical practice; that is, PVAS and physician's global assessment (PGA) (Figure 4). Pronounced (positive and inverse) correlations among all measured variables were observed in PR3-AAV (Figure 4C) but not in MPO-AAV (Figure 4B) or ANCA-negative vasculitis (Figure 4A). In PR3-AAV correlations between the inflammatory markers and at least either of the two disease activity scores (PVAS and PGA) were notable for all inflammatory markers except CRP (Figure 4C). Following statistical correction for multiple correlations, associations between PVAS and PGA, PVAS, and serum S100A12, PVAS/PGA, and ESR, S100A12 and PMN count as well as Hb and ESR proved significant.

In MPO-AAV, disease activity scored by PVAS or PGA had a pronounced negative correlation with Hb concentration (Figure 4B), while in ANCA-negative vasculitis no inflammatory markers correlated with PVAS or PGA (Figure 4A). In contrast and in line with findings shown in Figure 2, PMN counts correlate with S100A12 serum concentration in ANCA-negative vasculitis (Figure 4A) and PR3-AAV (Figure 4C) but not MPO-ANCA patients (Figure 4B).

## Discussion

Assessment of CPV disease activity at the time-of-diagnosis is not usually challenging in clinical practice with the most critical need for disease activity assessment coming after diagnosis, when disease is milder and clinical evidence of persisting activity or a flare following remission may be subtle. In this study, we assessed the potential contribution of inflammatory markers to disease activity scoring at times when therapeutic decisions would be made. Our analysis did not include any of the emerging vasculitis-subtype specific ‘activity' biomarkers (14) that were discovered in adult vasculitis by comparing patients with highly active vasculitis to patients in remission or to healthy controls (14). These markers have not been validated in independent cohorts and were not evaluated in patients with mild disease. For these reasons we restrict our analysis to inflammatory measures that are, or can be readily accessible, applicable to different phases of disease (active/inactive) and without requirements for sample preparations that are not compatible with routine clinical practice.

Our findings show that, for our cohort of 56 children with systemic CPV, classical laboratory measures of inflammation such as CRP, Hb, ESR, and PMN count as well as the neutrophil-derived protein S100A12 generally track with disease course from times of highly active disease at diagnosis, to milder disease following intensive therapy. Stratifying participants with small to medium sized vessel vasculitis (GPA, MPA, EGPA, PAN, and UCV) for the absence or presence of ANCA-specific antibodies, we find that inflammatory measures track more closely with disease course in individuals with PR3-AAV and to a lesser extent, ANCA-negative vasculitis. With the exception of Hb, inflammatory markers did not vary substantially with disease course in MPO-AAV. Most notably, we report higher serum concentrations of S100A12 and elevated PMN counts at the onset of disease in children with PR3-AAV compared to MPO-AAV that is not confounded by disease severity or corticosteroid therapy. Our data is consistent with previous reports showing that S100A12 correlated with inflammatory activity in Kawasaki disease, an acute form of vasculitis (18, 19). Further, inclusion of samples from children with Takayasu's arteritis, an ANCA-negative vasculitis affected large vessels in the heart did not significantly alter our findings (data not shown). These data suggest that our results may be applicable to forms of vasculitis that affect vessels of different size.

S100A12 is almost exclusively expressed by human neutrophils (17) and we observed consistent correlation of S100A12 serum levels with circulating neutrophils in ANCA-negative as well as PR3-AAV. This correlation is not observed in MPO-AAV. Similar to other inflammatory conditions (24) renal-limited MPO-ANCA vasculitis may involve more tissue-restricted inflammatory processes compared to ANCA-negative and PR3-ANCA CPV, which may depend on circulating or vessel-associated immune cell activation (4). Along similar lines, sequential changes in serum levels of S100A8/A9, a sister protein to S100A12, are correlated with mRNA expression in circulating granulocytes and monocytes in PR3-ANCA vasculitis. Additionally, higher concentrations of S100A8/A9 bound to endothelial cells was indicative of active disease (25), which has been similarly observed with S100A12 in giant cell arteritis (26).

Thus, we speculate elevated concentrations of serum S100A12 to be indicative of more systemic or necrotic inflammation in PR3- compared to MPO-AAV (4). A direct mechanistic link between high concentration of S100A12 and PR3-specific autoantibodies is not yet clear. However, the anti-angiogenic factor sFlt1 (also known as sVEGFR-1) is implicated in the pathogenesis of PR3-AAV by impairing endothelial cell repair. Monocytes were shown to serve as the main source of sFlt1 with release triggered by C5a and/or TLR4-ligation (27–29). Thus it may be conceivable that S100A12 in its capacity as an endogenous TLR4 ligand (30, 31) is able to trigger sFLT1 release and associate with PR3-reactive autoantibodies.

In summary, our observational study suggests that physicians should continue to measure and consider ESR, CRP, Hb and leukocyte count when assessing disease activity and more systematic and integrated use of these routine as well as new (S100A12) laboratory markers, together with clinical tools (PVAS, PGA) can assist and improve therapeutic decision making in the pediatric populations across vasculitis subtypes but most profoundly for subtypes such as GPA that are associated with PR3-ANCA. Given the rarity of CPV in children, our study enhanced enrollment by allowing participants to enter the study at any time point from diagnosis to remission. The result is a larger study cohort however collected samples for many individuals captured only a portion of disease course as opposed to the entire period from diagnosis to remission. Therefore, the current sample set was underpowered to assess the utility of laboratory markers for assessment of disease activity in individual patients as would occur in clinical practice. Our results require validation in a separate cohort that would then expedite uptake of findings into clinical care centers for the management of childhood systemic vasculitis.

## Author contributions

KB, DC, DF, and CK: study design; GA, KG, RH, CR, SB, RL, DC, and DF: data acquisition; KB, GA, DC, DF, and CK: data analysis; JL, KK, DL, and JG: statistical analysis; KB, DC, DF, and CK: manuscript draft. All authors manuscript revision.

### Conflict of interest statement

DF is participating in advisory boards of Chugai-Roche, Sobi, Novartis, and Pfizer and has received research grants from Novartis and Pfizer. RL has participated in advisory boards of Chugai-Roche, Grunenthal, GSK, and Medimmune and has received research grants from GSK and Roche. KK and DF have filed a patent relating to the use of proinflammatory S100A12 homomultimers in the diagnosis and treatment of inflammatory disorders (WO 2016/178154 A1). The remaining authors declare that the research was conducted in the absence of any commercial or financial relationships that could be construed as a potential conflict of interest.
